# A Novel Mechanism for Zika Virus Host-Cell Binding

**DOI:** 10.3390/v11121101

**Published:** 2019-11-28

**Authors:** Courtney A. Rieder, Jonathan Rieder, Sebastién Sannajust, Diana Goode, Ramaz Geguchadze, Ryan F. Relich, Derek C. Molliver, Tamara E. King, James Vaughn, Meghan May

**Affiliations:** 1 Department of Biomedical Sciences, College of Osteopathic Medicine, University of New England, Biddeford, ME 04005, USA; calexispearson@gmail.com (C.A.R.); jrieder@une.edu (J.R.); ssannajust@une.edu (S.S.); dgoode@une.edu (D.G.); rgeguchadze@une.edu (R.G.); dmolliver@une.edu (D.C.M.); tking6@une.edu (T.E.K.); jvaughn@une.edu (J.V.); 2 Center for Excellence in the Neurosciences, University of New England, Biddeford, ME 04005, USA; 3 Department of Pathology and Laboratory Medicine, Indiana University School of Medicine, Indianapolis, IN 46202, USA; rrelich@iupui.edu

**Keywords:** Zika Virus, Neurotropism, Flavivirus, Microcephaly, ASN154, N-acetylglucosamine, Encephalitis, binding motif

## Abstract

Zika virus (ZIKV) recently emerged in the Western Hemisphere with previously unrecognized or unreported clinical presentations. Here, we identify two putative binding mechanisms of ancestral and emergent ZIKV strains featuring the envelope (E) protein residue asparagine 154 (ASN154) and viral phosphatidylserine (PS). Synthetic peptides representing the region containing ASN154 from strains PRVABC59 (Puerto Rico 2015) and MR_766 (Uganda 1947) were exposed to neuronal cells and fibroblasts to model ZIKV E protein/cell interactions and bound MDCK or Vero cells and primary neurons significantly. Peptides significantly inhibited Vero cell infectivity by ZIKV strains MR_766 and PRVABC59, indicating that this region represents a putative binding mechanism of ancestral African ZIKV strains and emergent Western Hemisphere strains. Pretreatment of ZIKV strains MR_766 and PRVABC59 with the PS-binding protein annexin V significantly inhibited replication of PRVABC59 but not MR_766, suggesting that Western hemisphere strains may additionally be capable of utilizing PS-mediated entry to infect host cells. These data indicate that the region surrounding E protein ASN154 is capable of binding fibroblasts and primary neuronal cells and that PS-mediated entry may be a secondary mechanism for infectivity utilized by Western Hemisphere strains.

## 1. Introduction

Zika virus (ZIKV) is a mosquito-borne Flavivirus that recently emerged and established endemicity in the Western Hemisphere (reviewed here [1,2]). ZIKV disease historically presented as a mild febrile illness featuring myalgia, rash, and conjunctivitis. However, novel and more severe clinical presentations and increased disease incidence were reported as the virus emerged in the South Pacific [3,4] and the Western Hemisphere [5,6,7]. Since that time, case reports and animal models have implicated ZIKV in a congenital syndrome most notably featuring microcephaly [7,8,9,10,11,12], primary encephalitis, encephalomyelitis, lyssencephaly, or Guillan-Barré syndrome [5,13,14,15,16,17,18,19,20], chorioamnionitis [21], testicular infection [22], changes in semen quality [23], and potentially hemorrhagic shock syndrome [24,25]. The biology and pathogenesis of ZIKV were virtually unexplored at the time of its detection in the Western Hemisphere, making rapid progress toward diagnostics, therapeutics, or vaccine development challenging in the absence of viral targets [26]. Substantial progress in the understanding of ZIKV biology has been made in a short time and includes identification of potential host cell receptors [27,28,29]—factors that impact replication kinetics [30,31,32,33]—and the development of animals models for both neurological and prenatal disease [34,35].

Changes in clinical presentation during ZIKV infection from strains in the Asian/American lineage relative to the ancestral African lineage are likely the result of divergent nucleotide and amino acid sites conferring new phenotypes [36,37,38,39], and the relative contributions of various changes are still being explored. The structure of the ZIKV strain H/PF/2013 (Asian/American lineage) was resolved by cryo-electron microscopy and illustrated both unique features of this lineage and commonalities to other flaviviruses. A key difference between ZIKV lineages predicted computationally and detected structurally is the modification of asparagine at position 154 (ASN154) of the envelope protein (E) with N-acetylglucosamine (NAG). This modification is also seen across many flaviviruses but is often variable across strains [40]. Reports have implicated the ASN154 counterpart in host cell interactions by the four dengue virus serotypes [41] and in neuroinvasion of West Nile virus and St. Louis encephalitis virus [42,43]. Additionally, a recent study by Yuan et al. demonstrated that a single amino acid substitution within the PrM protein of Western Hemisphere strains conferred increased virulence and resulted in exacerbated pathology in vivo [33]. While this change confers an increased capacity for cell death and correlates with the clinical findings suggesting more severe and invasive disease, it cannot explain the newly emerged ability to directly invade the central nervous system (CNS). We sought to build upon our recent informatics analysis [36] by utilizing the findings to identify additional binding mechanisms of ZIKV. 

## 2. Methods

### 2.1. Virus Isolates and Culture Conditions

African Green monkey kidney (Vero) cells and Madin–Darby canine kidney (MDCK) cells were obtained from the American Type Culture Collection (ATCC, Manassas, VA). Cells were routinely propagated in Earle’s Minimum Essential Medium (EMEM) with Earle’s Balanced Salt Solution (BD Biosciences, San Jose, CA), supplemented with 10% fetal bovine serum, L-glutamine and Penicillin/Streptomycin. Cell cultures were incubated at 37 °C, with 5% CO_2_ and a relative humidity (RH) of 90%. Low-passage isolates of ZIKV strains MR_766 (ATCC VR-84, Uganda) and PRVABC59 (ATCC VR-1843, Puerto Rico) were obtained from the American Type Culture Collection. Virus stocks were propagated on monolayers of Vero cells. Harvested virus lysates were clarified by low-speed centrifugation (500× g/10 min) and stored in 1-ml aliquots at −80 °C. 

### 2.2. Protein Analysis and Peptide Design

The Envelope protein structure was visualized using Jmol via the Protein Data Bank (PDB ID 5JHM) [44,45], and the PDB Ligand Explorer was used to visualize the structure of NAG on ASN154. Probabilities of protein disorder at each amino acid site was estimated using PrDOS [46]. This analysis indicated that the region surrounding ASN154 constitutes a highly disordered linear epitope. Synthetic peptides representing this linear epitope including the differentially glycosylated ASN154 were generated (see Table 1) by Bachem (Bubendorf, Switzerland). The aminoterminal and carboxyterminal domains from PRVABC59 were also synthesized. Peptides were modified by the addition of an aminoterminal FITC label to allow detection and visualization.

### 2.3. Primary Dorsal Root (DRG) Ganglia Neuron Culture

Adult C57/black mice were anesthetized and perfused transcardially with 4 °C 1× PBS. Cervical, thoracic, and lumbar dorsal root ganglia (DRGs) were dissected in Ca^++^/Mg^++^-free Hank’s basic salt solution (HBSS) and dissociated as previously described [47]. DRGs were cultivated on laminin/polyD-lysine coated EZ slides (MilliporeSigma, Burlington, MA, USA) for 18–24 h in F-12 medium (Gibco, ThermoFisher Scientific, Waltham, MA, USA) supplemented with 10% fetal bovine serum, 1% penicillin/streptomycin at 37° C/5% CO_2_. Collection of DRG neurons was performed in accordance with a protocol approved by the University of New England’s Institutional Animal Care and Use Committee.

### 2.4. Peptide Binding Assays

Vero cells and MDCK cells, both of which are permissive for all Zika strains, were grown to 80% confluency in 48-well plates. Following the removal of medium, wells were blocked with 10% fetal bovine serum for 30 min at 37 °C. Peptides (0.2 μmol) were incubated with Vero or MDCK cells for 1 hour at 37 °C. Unbound peptides were removed by washing with 1× PBS, and mammalian cells were counterstained with 4′,6-diamidino-2-phenylindole (DAPI, diluted 1:300) to control for minor variations in monolayer populations. Bound peptides (FITC) were detected at 485/490 (excitation/emission), and cells were quantified at 350/460. Data are presented as FITC:DAPI ratios to correct for minor variance in cell numbers between wells. Statistical significance was measured by analyses of variance, and by Fisher’s Protected Least Significant Difference test for posthoc comparisons when main effects were significant (GraphPad Prism v. 6.0). 

Primary DRG neurons grown on coverslips were incubated with 100 µg peptide for 1 hour at 37 °C to qualitatively assess ZVBM binding potential. Unbound peptides were removed by washing with HBSS, and DRG neurons were counterstained with DAPI (diluted 1:300). Bound peptides were visualized using Keyence BZX-700 inverted widefield digital microscope. Binding was evaluated qualitatively to avoid ambiguities from the potential for greater or lower affinities for certain peptides by heterogeneous populations of neurons that may vary slightly from well to well, and from the formation of cellular islands as opposed to confluent monolayers. To avoid ambiguities with the impact of glycosylation, peptides PRV, MR, and PRV^Scr^were used.

### 2.5. Viral Inhibition Assays by ZVBM Peptides

Vero cells were propagated in 48-well plates (seed concentration-1e5 cells/well) for 24-h. Resulting monolayers (85% confluence) were washed twice with warmed PBS and incubated for 2 hours (37 °C, 5% CO_2_, 90% RH) with 0.1 ml volumes of either PBS (Negative Control), or PBS containing 0.8 μmol of the selected ZVBM peptide. Following treatment, PBS or peptide was decanted, and monolayers washed twice with warmed PBS. ZIKV stocks (stock concentrations: Strain MR_766–10^7^ TCID_50_/ml; Strain PRVABC59–10^7^ TCID_50_/mL) were serially diluted in serum-free Dulbecco’s Minimum Essential Medium (DMEM). Host cell monolayers in treated, or untreated plates were inoculated with either strain MR_766 or PRVABC59 (0.1 mL/well, 5 wells per dilution [MOI = 1], N = 3 replicates each) and incubated for 2 hr. After inocula were removed, wells were supplemented with 0.5 ml EMEM growth medium and returned to the incubator. Virus cytopathogenic effects (CPEs) were monitored and scored over a period of 10–12 days and the resulting virus titers calculated as TCID_50_/mL. Statistical significance of changes in virus titer as a result of peptide pretreatment versus untreated control was measured by *Student’s T*-test (GraphPad Prism v. 6.0). 

### 2.6. Virus Treatment with Annexin V

Annexin V (AbCam, Cambridge, MA) was dissolved in PBS (2335 μg/mL) and filter-sterilized (0.2 μm). ZIKV stocks MR_766 and PRVABC59 were then serially diluted in either PBS, or PBS-Annexin V. Dilutions were incubated for 2 hours (37° C, 5% CO_2_, 90% RH). Host cell monolayers, prepared in 48-well plates as previously described, were inoculated with respective virus dilutions (0.1 mL/well, 5 wells per dilution [MOI = 1], *n* = 3 replicates each). Virus CPE were scored over a period of 10–12 days and the resulting virus titers calculated as TCID_50_/ml. Statistical significance of changes in virus titer as a result of Annexin V pretreatment versus untreated control was measured by *student’s T* test (GraphPad Prism v. 6.0) for each ZIKV strain. 

## 3. Results

### 3.1. Binding Motif Prediction

**Figure 1:**
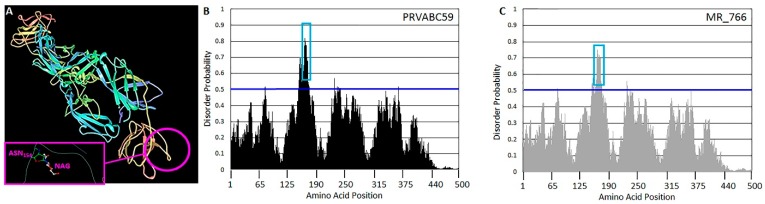
Envelope protein structure. (**A**) The predicted E protein structure indicates that the region containing ASN154 (circled) and its N-acetyl glucosamine (NAG) modification (inset) is a linear β strand. Intrinsic disorder probabilities were calculated for each amino acid position in the E protein sequence from strains (**B**) PRVABC59 and (**C**) MR_766. Probabilities above 0.5 (blue line) are considered indicative of sites representing disordered regions. The region containing ASN154 is indicated (blue box) for each strain.

Structural modeling predictions of strain PRVABC59 (Puerto Rico, 2015) indicated that asparagine 154 (ASN154) is part of a linear β strand (Figure 1A). The disorder probability of this region peaks at 0.72 (Figure 1B), suggesting that this portion of the E protein is particularly dynamic and flexible. Structural and disorder probability predictions of the African type strain MR_766 (Uganda 1947) exhibit similar characteristics (Figure 1C). This region was termed the (putative) Zika virus binding motif (ZVBM). ZVBM sequences from strains PRVABC59 and MR_766 were synthesized and N-terminally labelled with fluorescein isothiocyanate (FITC) in order to assess their capacity to bind ZIKV-susceptible and -permissive cell lines, disrupt ZIKV adsorption, and to interact with dorsal root ganglia (DRG) neurons *ex vivo*. The PRVABC59 sequence was used to generate a peptide that was modified with an N-acetyl glucosamine (NAG) molecule at position 8 (equivalent to ASN154), as it natively occurs in this strain, and without carbohydrate modification (Table 1).

### 3.2. ZVBM Binding to ZIKV Replication-Permissive Fibroblast Lines

**Figure 2:**
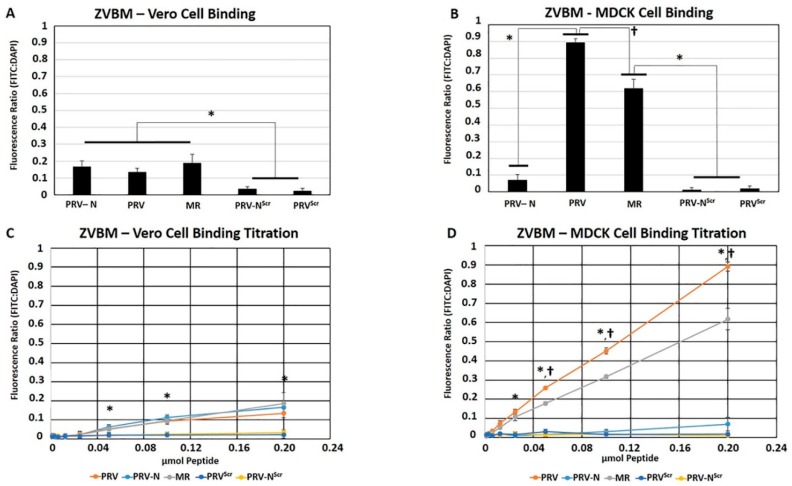
Zika virus binding motif (ZVBM) binding and Zika virus (ZIKV) inhibition in Vero cells. Peptides PRV, PRV-N, and MR bound Vero cells significantly (* *p* < 0.05) above scrambled PRV^Scr^ and PRV-N^Scr^ controls (**A**). Peptides PRV and MR bound Madin–Darby canine kidney (MDCK) cells significantly (* *p* < 0.05) above PRV-N and scrambled PRV^Scr^ and PRV-N^Scr^ controls, and PRV bound with significantly († *p* < 0.05) higher avidity than MR (**B**). Two-fold dilutions of ZVBM peptides resulted in proportional reductions in signal, with significant (*, *p* < 0.05) differences between binding peptides and scrambled controls apparent with 0.05 μmol for Vero cells (**C**) and 0.0125 μmol for MDCK cells (**D**). The difference in avidity between PRV and MR became significant († *p* < 0.05) with 0.0125 μmol of treatment. Error bars indicate standard deviations in all panels.

ZVBM peptides from strains MR_766 (“MR”) and PRVABC59 (NAGylated and unglycosylated, “PRV” and “PRV-N”) all bound Vero cells at levels significantly above those of scrambled (NAGylated and unglycosylated, “PRV^Scr^” and “PRV-N^Scr^”) controls (Figure 2A). Unglycosylated ZVBM peptides MR and PRV both bound Madin–Darby canine kidney (MDCK) cells significantly (*p* < 0.05) above scrambled controls PRV^Scr^ and PRV-N^Scr^, though the signal generated by PRV was significantly higher than MR. Interestingly, NAGylated PRV-N did not bind MDCK cells above the scrambled controls, (Figure 2B). Two-fold dilutions of adherent ZVBM peptides resulted in proportional reductions in FITC signal, indicating that titration of binding to both Vero and MDCK cells is readily achieved (Figure 2C,D).

### 3.3. ZVBM Binding to Primary Neuronal Cells Ex Vivo

**Figure 3:**
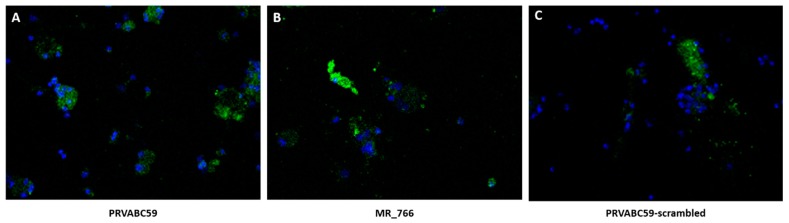
Peptide binding to dorsal root ganglia (DRG) neurons ex vivo (20x magnification). Primary DRG neurons from C57 black mice (DAPI, blue fluorescence) were exposed to ZVBM peptides (FITC, green fluorescence) from (**A**) PRVABC59, (**B**) MR_766, and (**C**) scrambled (unglycosylated) PRVABC59. Punctate green staining around the DRG nuclei was observed in panels A and B, but was largely absent from panel C.

We collected dorsal root ganglia (DRG) from C57/black mice and cultured DRG neurons on coverslips to qualitatively assess interactions with ZVBM peptides. Peptides PRV and MR were visualized in association with 24-hour DRG neuron cultures by fluorescence microscopy (Figure 3A–B). Cell association was not detected for the scrambled PRVABC59 control peptide (Figure 3C).

### 3.4. Refinement of ZVBM Functional Elements

**Figure 4:**
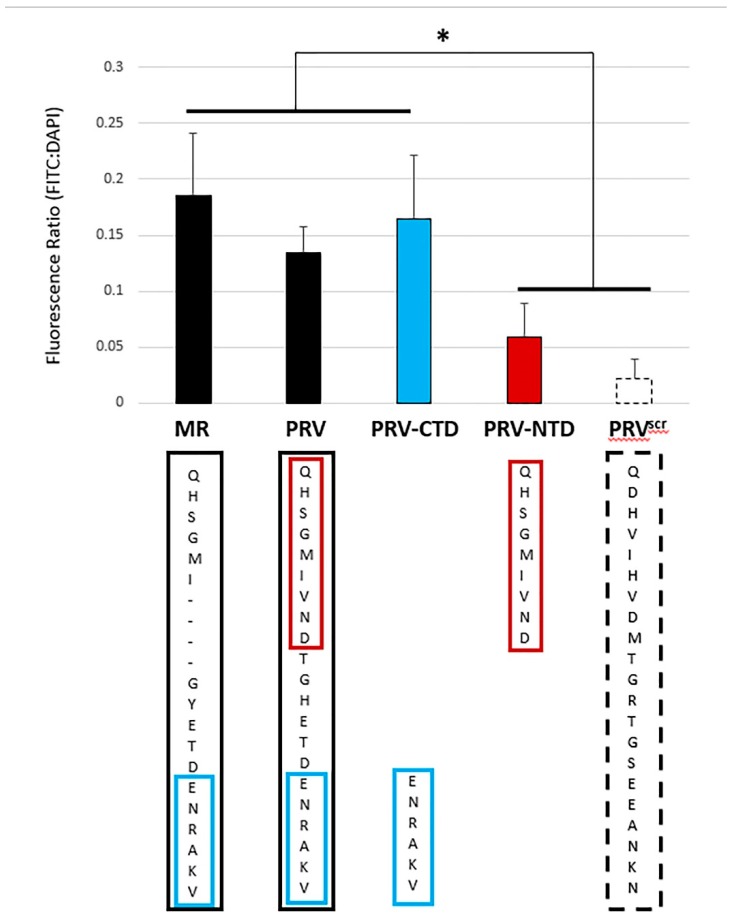
Refinement of ZVBM functional elements. The carboxyterminal peptide PRV-CTD (blue) bound Vero cells significantly (* *p* < 0.05) above the scrambled control peptide PRV^Scr^ (white), and the aminoterminal peptide PRV-NTD (red) did not. PRV-CTD bound Vero cells at equivalent levels to peptides PRV-N, and MR (black), indicating that this refined motif facilitates binding to Vero cells.

Peptides representing the NTD and the CTD of strain PRVABC59′s ZVBM sequence (“PRV-NTD” and “PRV-CTD”) were synthesized and N-terminally labelled with FITC (Figure 4). To avoid ambiguities with the impact of glycosylation, neither peptide was NAGylated, and Vero cells were used to assess binding. PRV-NTD was unable to bind Vero cells above the scrambled control PRV^Scr^, whereas PRV-CTD bound significantly (*p* < 0.05) above PRV^Scr^. Additionally, PRV-CTD bound at equivalent levels to full-length PRV and to MR.

### 3.5. Disruption of ZIKV Infection and CPE Generation

**Figure 5:**
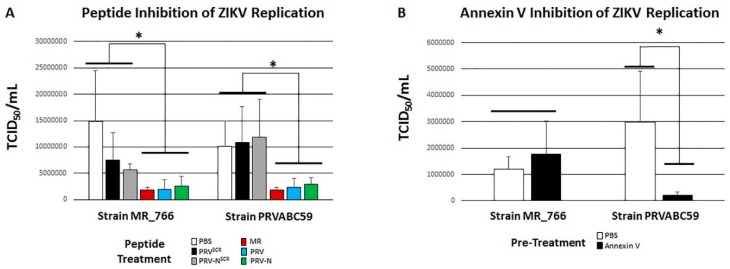
Disruption of ZIKV Infectivity. Pretreatment of Vero cells with peptides MR (red), PRV (blue), and PRV-N (green) significantly (* *p* < 0.05) inhibited cytopathogenic effect (CPE) generation following infection with both strains MR_766 and PRVABC59 relative to pretreatment with scrambled controls PRV^Scr^ (black), PRV-N^Scr^ (grey), or PBS alone (white) (**A**). Pretreatment of ZIKV strain PRVABC59 with the PS-binding protein annexin V (black) prior to Vero cell infection resulted in a significant (* *p* < 0.05) decrease in CPE generation relative to PBS alone (white). Pretreatment of ZIKV strain MR_766 with annexin V did not impact CPE generation relative to pretreatment with PBS alone (**B**).

Host cell monolayers were pre-treated with PRV, PRV-N, MR, and scrambled controls PRV^Scr^ and PRV-N^Scr^. To avoid ambiguities from the impact of glycosylation, Vero cells were used to for this analysis. All peptides were used to inhibit both strains MR_766 and PRVABC59. Pretreatment of Vero cell monolayers with PRV, PRV-N, and MR significantly (*p* < 0.01) inhibited infectivity and CPE generation by both ZIKV MR_766 and ZIKV PRVABC59 relative to scrambled control peptides and Vero cells pretreated with PBS alone (Figure 5A). MDCK cells are still permissive for ZIKV replication [48], suggesting that additional mechanisms facilitate infection when ZVBM is NAGylated. We pretreated ZIKV strains MR_766 and PRVABC59 with the PS-binding protein annexin V prior to infection of Vero cell monolayers. Annexin V significantly (*p* < 0.05) inhibited infectivity of PRVABC59 relative to untreated controls but did not inhibit MR_766 (Figure 5B).

## 4. Discussion

The change in clinical spectrum of ZIKV disease caused by Asian/American lineage strains suggests fundamental changes in key protein functions relative to African lineage strains. Comparative analyses between strains of both lineages identified areas of interest with potential to contribute to the changes in clinical presentations, including ASN154 [36,40]. ASN154 is part of an intrinsically disordered region of the E protein. Disordered regions lack fixed tertiary structures, which affords them flexibility in spatial arrangement. The array of structures expands further if the disordered regions are post-translationally modified, including via glycosylation [49]. The diversity that this provides in terms of function is increasingly reported for many organisms, including flaviviruses [50,51]. The region surrounding ASN154 was thus an attractive target for mechanistic evaluation to explore the changes in clinical presentation.

ZVBM peptides MR, PRV, and PRV-N all bound Vero cells at levels significantly above those of scrambled controls (Figure 2A), and peptides MR and PRV bound MDCK cells significantly above scrambled controls (Figure 2B). Interestingly, NAGylated PRV-N did not bind MDCK cells above the scrambled controls, (Figure 2B). Two-fold dilutions of adherent ZVBM peptides resulted in proportional reductions in FITC signal, indicating that titration of binding to both Vero and MDCK cells is readily achieved (Figure 2C,D). These findings suggest that this motif binds to fibroblasts and has the potential to mediate ZIKV infectivity, but the difference in avidity between PRV and MR and the inability of NAGylated PRV-N to bind MDCK cells indicates that the functionality of ZVBM may be host cell and strain dependent. Pre-treatment of host cell monolayers with PRV, PRV-N, MR significantly inhibited infectivity and CPE generation by both ZIKV MR_766 and ZIKV PRVABC59 relative to scrambled control peptides and Vero cells pretreated with PBS alone (Figure 5A), suggesting that ZIKV and ZVBM target the same host cell receptor. These findings demonstrate that adherence of ZVBM peptides to Vero cells has functional relevance, and that this motif likely mediates at least some host cell infection.

Binding of the MR_766 ZVBM peptides to Vero cells and MDCK cells, despite a four-amino acid deletion relative to PRVABC59, suggests that the critical portion of the ZVBM is potentially contained entirely on the aminoterminus (NTD) or the carboxyterminus (CTD). PRV-NTD was unable to bind Vero cells, whereas PRV-CTD bound significantly (*p* < 0.05) above scrambled controls. Consistent with the notion that binding is facilitated by either the NTD or the CTD, PRV-CTD bound at equivalent levels to full-length PRV and to MR indicating that the functional element of ZVBM that mediates binding is contained entirely on the CTD (Figure 4). Interestingly, PRV-CTD does not contain ASN154, suggesting that any impacts of this residue on host cell binding and/or specificity stem from its proximity to the critical binding mediators rather than its direct involvement.

Given that MDCK cells are still permissive for ZIKV replication [48], we hypothesized that a more generalized mechanism may be contributing to viral adsorption to some strains. The association of human AXL with ZIKV adsorption [27,28,29] suggests that viral PS may facilitate entry into certain host cells by binding Gas6, which in turn binds AXL, as is seen with multiple viruses [52,53,54,55,56]. Pretreatment ZIKV strain PRVABC59 with the PS-binding protein annexin V prior to infection of Vero cell monolayers significantly inhibited infectivity relative to untreated controls. Conversely, pretreatment of ZIKV strain MR_766 with annexin V did not impact infectivity (Figure 5B). This finding suggests that PS-mediated ZIKV adsorption is possible for at least some strains, although a relationship between PS-mediated infectivity and ZVBM- NAGylation is unclear, and it is plausible that strains differing in maturation kinetics will differ in membrane PS concentration. While at least one additional mechanism has been described for the greater infectivity of Asian and American strains [33], PS-mediated host cell entry has high potential to contribute to this phenotype as well. Additionally, these findings also support previous studies that both implicate AXL as a host cell receptor for Asian/American ZIKV strains and those that show genetically ablated animals are still susceptible to infection by establishing two distinct binding mechanisms for this clade [27,28,29,57,58,59].

CNS disease or infectivity with MR_766 following intrathecal or intracerebral inoculation *in vivo*, or neuronal cell infectivity in vitro, has been reported [60,61,62,63,64]. Observation of the MR peptide binding to DRG neurons is consistent with these findings; however, both stand in conflict with a lack of evidence for CNS involvement during human disease caused by African ZIKV strains. We hypothesized that exposure to neuronal cells ex vivo would result in MR_766 ZVBM peptide binding, and the lack of neurological complications during Zika virus disease caused by African strains stems from an inability of these strains to penetrate into the CNS. These findings were consistent with those of Annamalai et al., who demonstrated a lack of neurological disease with strains lacking NAGylation at ASN154 when injected intravenously, and overt disease when the same strain was injected intracranially [65]. This suggests that the ability of Asian/American lineage strains to cause disease in the CNS is not exclusively due to a novel binding mechanism but is more likely due to a newfound ability to access the CNS and other privileged body sites. The ZVBM peptide MR binding to neuronal cells ex vivo is consistent with this suggestion. While additional mechanisms facilitate novel interactions between Asian/American lineage ZIKV strains and neuronal cells [33], ZVBM-mediated binding to neuronal cells is not unique to this lineage. N-linked glycosylation of E in other flaviviruses is proposed to facilitate blood-brain barrier (BBB) transit by allowing interaction with DC-SIGN on microvascular endothelial cells followed by transcytosis [66], and ASN154 is notably absent from strain MR_766. Though not required for direct binding to neuronal cells, ASN154 may very well facilitate BBB passage in Asian/American lineage strains. In vivo inhibition of Asian/American ZIKV BBB transit by peptides would demonstrate this.

The outcomes of our in vitro and ex vivo studies suggest that the disordered region of the ZIKV E protein surrounding ASN154, termed ZVBM, binds fibroblasts and primary neuronal cells, indicating its potential to contribute to host cell infection. The carboxyterminal portion of the ZVBM (i.e., ENRAKV) is capable of binding fibroblasts at the same levels as full-length ZVBM, suggesting that it mediates the binding. Despite retaining the sequence ENRAKV, glycosylated peptides have differing abilities to bind MDCK cells and ZIKV strains lacking ASN154 feature different tissue tropism, suggesting that glycosylation of this site could either sterically hinder binding to some cell types, provide proximity to other cell types by facilitating entry into the privileged body sites, or both. This model is consistent with both the clinical disparity between ZIKV lineages and the generation of neurological disease by the African strain MR_766, which lacks ASN154, when introduced directly into the CNS as previously described [61,65]. These findings demonstrate the impact of NAGylation of a pathogen surface protein in the vicinity of a binding motif on its potential host cell targets. This change in post-translational modification can therefore instantly alter the potential target tissues of infectious agents and can be expected to similarly alter the array of clinical presentations they cause in turn. This concept has widespread implications for parallel changes in other pathogens, and therefore applies broadly to the field of emerging infectious diseases.

## Figures and Tables

**Table 1:** Peptide sequences.

Peptide Name ^a^	Strain	Sequence ^b^	Strain Type ^c^	Molecular Weight
PRV-N	PRVABC59	*QHSGMIVNDTGHETDENRAKV	Asian/American	2949.082 g/mol
PRV	PRVABC59	*QHSGMIVNDTGHETDENRAKV	“African”	2727.872 g/mol
MR	MR_766	*QHSGMI----GYETDENRAKV	African	2324.482 g/mol
PRV^Scr^	N/A	*QDHVIHVDMTGRTGSEEANKN	N/A	2727.872 g/mol
PRV-N^Scr^	N/A	*QDHVIHVDMTGRTGSEEANKN	N/A	2949.082 g/mol
PRV-NTD	PRVABC59	*QHSGMIVND	“African” partial	1398.47 g/mol
PRV-CTD	PRVABC59	*ENRAKV	Asian/American	1105.192 g/mol

^a^ Peptide abbreviations represent the strain they derived from. The modifiers N, NTD, and CTD reflect the addition of NAG, use of the aminoterminal domain, or the carboxyterminal domain, respectively. The superscript “Scr” indicates a scrambled control peptide of the same designation. ^b^ Asterisk (*) indicate location of the FITC molecule. Shaded asparagine (N) residues indicate location of NAG coupling. ^c^ The designation “African” indicates that sequence from the Asian/American clade strain PRVABC59 has been made to resemble an African strain by its lack of NAG.

## Abbreviations

**ZIKV**	Zika virus
**E**	envelope protein
**PS**	phosphotidylserine
**CNS**	central nervous system
**BBB**	Blood-brain barrier
**ASN154**	Asparagine 154
**ZVBM**	Zika virus binding motif
**NAG**	N-acetylglucosamine
**FITC**	fluorescein isothiocyanate
**DAPI**	4′,6-diamidino-2-phenylindole
**NTD**	N-terminal domain (aminoterminus)
**CTD**	carboxyterminal domain (carboxyterminus)
**DRG**	dorsal root ganglion
**EMEM**	Earle’s minimal essential medium
**DMEM**	Dulbecco’s minimal essential medium
**HBSS**	Hank’s balanced salt solution
**PBS**	phosphate buffered saline
**TCID_50_**	50% tissue culture infective dose
